# Experiential Trajectories of Weight Gain: A Qualitative Study of People With Larger Bodies’ Understanding of Their Weight Changes Throughout Life in Norway

**DOI:** 10.1177/10497323251342217

**Published:** 2025-06-04

**Authors:** Yngvild Sørebø Danielsen, Vivian Woodfin, Signe Hjelen Stige

**Affiliations:** 1 Department of Clinical Psychology, 1658 University of Bergen, Bergen, Norway; 2 Regional Department of Eating Disorders, Haukeland University Hospital, Bergen, Norway; 3 Solli District Psychiatric Hospital, Bergen, Norway

**Keywords:** qualitative research, trauma, obesity, weight development, life experiences, hermeneutics, phenomenology

## Abstract

This qualitative study explores how people in Norway seeking treatment for ‘obesity’ experience and understand their weight development in a life-course perspective. Participants were adults (*N* = 10) who had recently attended a specialist lifestyle intervention. The study employed a hermeneutic phenomenological approach. Semi-structured in-depth life story interviews were conducted and were analyzed using reflexive thematic analysis and narrative perspectives. Results were categorized according to two dimensions: understanding versus it’s a mystery, and agency versus helplessness. Based on these two dimensions, four trajectories for experienced weight development were formulated: (1) “Snowballing weight gain” (understanding but limited agency); (2) “I see the path that leads here” (understanding and agency); (3) “Why me? Grasping at straws” (limited understanding and limited agency); and (4) “What happened? Making the best of it” (limited understanding but agency). Most participants found it hard to narrate the causes of their weight gain. The experience was not only that gaining weight was a problem but also narrating about a “problematic body” that is a part of who you are makes you both an object and a subject in the narrative. However, adverse life events and stress resulting in emotional eating were the most prominent themes presented. Agency was found to be impacted by trauma reactions, emotional pain, and repeated weight loss attempts. Trauma and emotional pain were at the core of our participants’ narratives about the causality of their weight gain, and more trauma-informed approaches are warranted for people with larger bodies in need of health care.

## Introduction

Merleau-Ponty in his phenomenological theory describes how we are present in the world and experience everything through our bodies (Merleau-Ponty, 2013). Living with a higher weight would in this perspective include experiencing all aspects of life through a larger body. In qualitative research exploring narratives about body size, the body becomes both an object and a subject—we both are and have bodies (Mol & Law, 2004). Narrating could alternate between experiencing the body as an object and not an integrated part of the self and the body being a reflexive tool to access important knowledge about the body itself, weight changes, and identity. In this view, how we relate to our bodies would influence both how we experience the world and have access to our own feelings and our experienced sense of self.

The individual narratives about the causality of weight gain and body size in the life stories of people with larger bodies are formed by these experiences of being in the world, as a part of society, in relationships and in meetings with health care workers and us as researchers (Warin & Gunson, 2013), all representing a variety of different understandings and narratives of body size and the causality of weight gain (Jebeile et al., 2022; Sholl & De Block, 2024).

Employing a narrative phenomenological–hermeneutical framework, our main aim in this qualitative study was to explore how people with larger bodies seeking treatment for ‘obesity’ understand and experience their weight development in a life-course perspective. How do they narrate about their weight history in the context of recent participation in a behavioral lifestyle intervention aimed at weight loss, and were there challenges in narrating about their weight development?

While the understanding and causality of ‘obesity’ or larger body size are negotiated and researched in the medical field and society, the insider perspective exploring people with larger bodies’ understanding of their weight development is scarce in research literature (Ekman, 2018; Farrell et al., 2021). A qualitative synthesis by Farrell et al. (2021) summarizing findings from individuals with larger bodies’ understanding of their weight gain identified only three studies on this topic (Bell et al., 2017; Owen-Smith et al., 2014; Smith & Holm, 2011). The most prominent causes put forward in these studies were early life experiences of trauma exposure, lack of love, negative emotional states, and eating for comfort (Farrell et al., 2021). Genetic factors, physical environments, and psychiatric medication were mentioned by some participants, but less frequently than the psychological aspects (Farrell et al., 2021). Grannell, le Roux, et al. (2021) likewise found that few patients interviewed at a tertiary ‘obesity’ clinic mentioned biological determinants when asked about their beliefs on the causality of having a larger body, while lifestyle, diet, and limited exercise were the most prominent causes reported, together with adversities resulting in emotional eating. To summarize, the most prominent causality stories about having a larger body from these qualitative studies are about the function of food in regulating or numbing emotions caused by traumatic relational experiences.

A few recent studies from treatment contexts have more broadly explored *how* people with larger bodies narrate about experienced causes of weight gain. A Swedish study from a group treatment setting found that the participants showed a strong need “to explain why they had ended up where they were (having a larger body)” while this was not the explicit topic of their study (Imhagen et al., 2023). The researchers describe the interviews as an ambivalent process in which the participants expressed a sense of personal responsibility for gaining weight, while at the same time a need to explore or justify why. Some of their participants also explicitly stated that they found it hard to figure out or narrate about why they had gained weight (Imhagen et al., 2023). These findings illustrate the challenging narrative process of being both a subject and an object when exploring body size, as well as what could be interpreted as a need to defend oneself against weight stigma related to the individual responsibility framing of body size. Another qualitative study from the United Kingdom likewise highlighted how people undergoing bariatric surgery narrated their weight story to resist the construction of their higher body weight as being an individual failure. The paper discusses how their stories might be shaped as a response to societal views framing ‘obesity’ as a lack of willpower and motivation for acquiring healthy habits (Throsby, 2007). These findings show the tension between experienced powerlessness and feelings of responsibility when narrating life with a larger body. Ekman (2018) explored how people formulated their explanations for having a larger body despite repeated weight loss attempts. She introduced the terms “cause and problem shifting.” The terms refer to participants narrating overeating as grounded in underlying problems or being a symptom of something else not addressed in the weight loss interventions. The narratives engaged most vividly in exploring the functional aspects of eating, such as dealing with painful feelings or filling an existential void. Further, the participants found the ‘obesity’ interventions or diets contributing to their problem by being impossible to adhere to and finally resulting in loss of control, or the diet reprogramming biological mechanisms enhancing weight gain. Despite this, self-blame and personal responsibility framings were part of all the narratives (Ekman, 2018).

Further, Luig et al. (2020) have studied how people with larger bodies may negotiate and change their narrative about weight, body, self, and coping through clinical dialogues with health care professionals. They highlight how this co-creation of new narratives could lead to transformative moments, agency, and tangible health outcomes for the individuals (Luig et al., 2020). This co-creating of narratives would also take place during qualitative research interviews exploring understandings of weight gain and related factors, although taking a more explorative and less intervening form. Warin and Gunson (2013) discussed how in qualitative studies “cultural meanings associated with obesity are silenced and negotiated in the research process” and also how the relationship between the bodies of the informant and researcher might impact the communication and meaning-making. They argue that the limited amount of qualitative research about the experiences of living with larger bodies might partly be due to the challenges researchers face in conducting these studies (Warin & Gunson, 2013). A practical consequence of their discussion could be the need for reflexivity and not becoming a silent and invisible researcher in qualitative studies on weight and body size.

### Different Framings of Body Size

Individual narratives are always, to some degree, formed as a response to the surroundings of the people exploring their experiences and views. Therefore, some prominent discourses in society about body size and ‘obesity’ are relevant for analyzing and interpreting findings in this qualitative study and are presented briefly below.

#### The Longstanding Individual Responsibility Framing of Weight Gain

The ‘obesity’ discourse in society, dieting literature, and media has a long tradition of framing ‘obesity’ as an imbalance between energy intake and expenditure resulting from unhealthy eating and activity behaviors (Ferreira & Webber, 2022; Westbury et al., 2023). A recent study published in the Lancet found that 80% of 5623 people interviewed believed that ‘obesity’ could be cured by changing lifestyle behaviors (O’Keeffe et al., 2020). Consequently, living with a larger body would be perceived as an individual failure to adhere to healthy lifestyle behaviors caused by a lack of knowledge, motivation, or willpower (Ekman, 2018; Spratt et al., 2023). This conceptualization of body size as being under voluntary control has shaped societal views on body size for decades and contributed to stigma toward people with larger bodies (Spratt et al., 2023; Westbury et al., 2023). The individual responsibility framing also fits well with general trends in Western societies making the body a project to be perfected with super food, personalized workouts, and mental training, and there are large commercial interests in offering products and “solutions” advertised as producing weight loss (Ekman, 2023; Ferreira & Webber, 2022; Yeo, 2021).

Behavioral lifestyle interventions have been the recommended and most widely offered treatment for ‘obesity’ prior to the introduction of newer medications (GLP-1 analogues) (NICE, 2014). These interventions rely on improving knowledge about healthy habits together with supporting changes in eating and physical activity to produce weight loss. In several ways, they are communicating that weight loss should be possible with individual motivation and efforts put into behavior change. Although generally effective for weight loss in the short term, behavioral lifestyle interventions have proved limited utility in supporting long-term weight reduction (Nordmo et al., 2020). Several researchers and people with lived experiences argue that these treatments could be more harmful than helpful because they are constructing individuals as failing and contributing to weight stigma (Ekman, 2018; Puhl & Heuer, 2010). Ekman (2018) highlights how follow-up studies framing their research as to why individuals “fail to maintain weight loss over time” are blaming the individuals for not managing long-term weight stability rather than questioning the usefulness of the interventions they have participated in.

#### Current Clinical Theories About ‘Obesity’

During the last decade, health organizations have reached a broad consensus in defining ‘obesity’ as a multifactorial, chronic, relapsing non-communicable disease (Busetto et al., 2024; ObesityCanada, 2024; Rubino et al., 2025). The American Medical Association classified ‘obesity’ as a disease already in 2013, with the goal of reducing stigma and improving treatment (Pollack, 2013; Schumacher et al., 2023), moving away from the individual responsibility framing. The Lancet commission for the definition and diagnostic criteria of clinical obesity recently published new guidelines for defining clinical ‘obesity’ by symptoms resulting “from the effect of excess adiposity on the function of organs and tissues,” rather than BMI (Rubino et al., 2025). However, stakeholder organizations have been criticized for not providing clear-cut explanations for why ‘obesity’ is a disease. In other words, what are the mechanisms and causes of this disease (Grannell, Fallon, et al., 2021; Hofmann, 2016)? Obesity Canada’s guidelines have been at the forefront in describing ‘obesity’ as a genetic brain-related disorder affecting appetite regulation (ObesityCanada, 2024), building on neuroscience studies finding that gaining weight beyond a certain point impacts sub-cortical structures in the brain that regulate hunger, satiety, and weight (Grannell, Fallon, et al., 2021). These brain areas are sub-conscious and cannot be controlled by cognitive or behavioral effort (Grannell, Fallon, et al., 2021). This research and understanding of mechanisms maintaining higher weight are posing a challenge to the assumption that body weight to a large degree is under voluntary control (Sholl & De Block, 2024; Yeo, 2021). Still, the usefulness versus harmfulness of medical definitions of ‘obesity’ remains under debate (Ferreira & Webber, 2022; Sholl & De Block, 2024), as ‘obesity’ stigma in itself is detrimental to health (Puhl & Suh, 2015; Remmert et al., 2019).

#### Critical Fat Studies’ Attention to Stigma and Medicalization

Critical fat scholars analyze weight stigma in a sociological context highlighting how medical societies have contributed to producing stigma by defining the larger body, and thus people with larger bodies, as a problem that threatens society (e.g., by economic burdens on health care or not contributing to society) (Ferreira & Webber, 2022). The longstanding reinforcement of a lifestyle understanding of ‘obesity’ implying individual responsibility for body size is central to this stigma (Ekman, 2018; Rich & Evans, 2005). The castigation of the larger body as problematic in medicine is also seen as difficult to disentangle from sexist body ideals, moral healthism, and ordering people’s value by body size (Ekman, 2023; Ferreira & Webber, 2022). With regard to determinants of body size, the critical fat scholars draw attention to genetic and biological factors, but are specifically aware of how minority stress, stigma, and socio-economic factors contribute to weight gain (Ferreira & Webber, 2022; Leonard et al., 2024; Rich & Evans, 2005). Likewise, they describe how repeated weight loss attempts, outside and within lifestyle weight loss treatments, could be a risk factor for developing eating disorders and further weight gain (Greenhalgh, 2016; Pietiläinen et al., 2012). The critical fat scholars are aspiring to “demedicalize bodies and empower those who are marginalized through pathology by reclaiming their spaces and voices” (Warin & Gunson, 2013).

Qualitative studies exploring the lived experiences of people with larger bodies in line with this highlight the negative impact of weight stigma (Haga et al., 2020; Thomas et al., 2008; Ueland et al., 2019). Lewis et al. (2011) found that the people with larger bodies interviewed in their study from Australia rarely stood up to stigma but rather blamed themselves for being stigmatized, and the participants narrated how they experienced stigma impacted their emotional health and reduced their sense of self-worth and agency.

As summarized above, the insider perspective of people living with larger bodies is mainly lacking in research on the causality of weight gain and body size, and more qualitative studies are warranted. Our study contributes to literature by interviewing people living with larger bodies and seeking lifestyle treatment about how they experience and understand their weight development in a life-course perspective, and which life experiences and factors they consider pertinent for their weight development throughout life. It also discusses how the causality of weight and body size is narrated in the life stories of the individuals, in relation to their experiences from health care and to societal views on determinants of body size.

## Background

Our study included people that had recently participated in a behavioral lifestyle intervention for ‘obesity’ in Norway. Behavioral lifestyle interventions are often recommended as standard first-line treatment for ‘obesity’ (NICE, 2014). The behavioral lifestyle interventions aim at helping the participants improve diet quality and eating habits and be more physically active by reducing stimulus-driven behavior and promoting goal-directed intentional behaviors (Michaelsen & Esch, 2023). Most interventions include education about healthy habits, goal setting, planning, self-monitoring, problem solving, challenging dysfunctional thoughts and triggers for unhelpful behaviors, and conducting behavioral change (NICE, 2014). This treatment relies on an understanding that ‘obesity’ may be reduced by learning and integrating new lifestyle behaviors. In the treatment that our participants were enrolled in, weight loss of 5%–10% over a 2-year period was put forward as a possible goal, while changing habits were the focus in the session-to-session follow-up. They participated in 10 weekly group sessions and further individual follow-up for 2 years (on average four sessions a year).

## Methods

### Theoretical Framework

This study employs a phenomenological–hermeneutical approach to explore how adults seeking treatment for ‘obesity’ experience and understand their weight development during their life-course. In this paradigm, knowledge is seen as a construction and co-creation between informants and researchers, and subjective experiences and interpretations are at the core (Hatch, 2002). In line with a phenomenological focus, we employed life story interviews, allowing participants to dwell with and elaborate on their experiences with and perspectives on the phenomena under study. However, acknowledging the situated nature of knowledge and the inevitable influence of interpretation in all human activity, a dialogical view of reflexivity was central to the research process (Alvesson & Sköldberg, 2000; Angen, 2000; Laverty, 2003; Stige et al., 2009) (see also more details below).

### Recruitment and Participants

Inclusion criteria for the study were age >18 years, having participated in lifestyle treatment for “severe obesity” (defined as BMI >35 or BMI >30 with health complications), and being able to give informed consent. There were no specified exclusion criteria.

Based on previous research employing thematic analysis of life story interviews, we estimated that we required a minimum of 10–12 interviews to reach theoretical saturation for analysis (Saunders et al., 2018; Woodfin et al., 2021). Project assistants gave information about the study at lifestyle treatment at a specialist ‘obesity’ clinic at Haukeland University Hospital in 2019–2020. Eleven people made contact for participation and received written information about the study. They were subsequently contacted by phone for more elaborate project information and scheduling of interviews. Ten of the 11 agreed to participate and signed the informed consent form. After the 10th interview, we found we had reached sufficient breadth and depth in the emerging themes within the data material and concluded recruitment.

Four men and six women were interviewed. This matches the percentages of referrals to the ‘obesity’ outpatient clinic of 63.4% women. The youngest participant was in her 20s, while the oldest was in his 50s (mean age 43 years). The mean BMI was 41.3 (34.0–46.5). Eight participants lived with a partner or were married, and four participants had children. Five participants had an education of <3 years after high school. Eight participants were employed full time, while two received a disability pension. All participants, as well as the interviewer, were born in Scandinavia and had a Caucasian ethnicity, even though some had lived abroad for several years.

### Data Collection

Demographic information and BMI were self-reported. The individual semi-structured in-depth interviews were conducted at the Department of Clinical Psychology at the University of Bergen by the first author who is a psychologist. Maximum timeframe for the interviews was three hours. Most of the interviews lasted approximately two hours. The interviewer presented herself as independent from the treatment setting at the start of the interview to underline that the participants could freely share their experiences from the treatment without being afraid to hurt anyone. The interviewer also shared that she had personal experiences from developing and living with “overweight,” to facilitate a safe setting to talk about weight and body where the word “overweight” was not silenced implying that it is something that you should be ashamed of. The interviewer strived to openly explore and engage in the narratives of the participants, validating their experiences and normalizing when found appropriate. We wanted to explore the individuals’ experiences with and understanding of their weight development and how they give meaning to this phenomenon in their lives. We therefore chose the Life Story Interview by Dan P. McAdams as a starting point when developing the interview guide (McAdams, 2008). Questions were modified to explore the participants’ experiences and meanings related to their weight development and other important life events. The interview included many broad questions that invited the participant to narrate freely about experiences and thoughts. The interview guide was also open to follow-up questions from the interviewer to facilitate exploration of meaning given to the phenomena (Interview Guide, Appendix A).

The interviews were audiotaped and transcribed verbatim by psychology students at the University of Bergen.

### Researcher Position and Reflexivity

YSD conducted the interviews, and analyzed and drafted the paper. She is a clinical psychologist specializing in eating disorder treatment and has been working as a clinician and researcher in the ‘obesity’ field since 2006. She is between 40 and 50 years, Norwegian by nationality and ethnicity, and has a BMI fluctuating between the ‘overweight’ and ‘obesity’ categories. When starting to work in an ‘obesity’ clinic as a psychologist, she was struck both by the patient’s stories of adverse experiences and by the lack of focus on mental health, trauma reactions, and eating disorders in the follow-up of patients with “severe obesity.” She holds an agnostic view as to whether these psychological factors should be considered causes of ‘obesity’ but holds the opinion that they clearly influence body perceptions, eating habits, and quality of life in people living with larger bodies, while genetic predisposition might be more important for the development of ‘obesity’.

Sharing the view on ‘obesity’ as a biological disease of appetite regulation, making long-term weight stabilization demanding, she still considers psychological and behavioral treatment supporting healthy life habits (including mental health and self-compassion) as an important component in ‘obesity’ health care. While long-term weight loss is not perceived as a realistic goal of these interventions, promoting body acceptance, healthier life habits and coping skills, realistic weight goals, better quality of life, and reduced risk of eating disorders could, in her perspective, be viable outcomes. And while newer ‘obesity’ medications appear to be a game changer in ‘obesity’ medicine, her view is that medications should be a part of a more holistic long-term follow-up. She acknowledges that in practice, lifestyle interventions are not always designed and delivered in a manner that promotes the outcomes described above and could be experienced as setting treatment-seeking people with larger bodies up for failure or not addressing their experienced needs. She finds the fight against stigmatization of people living with larger bodies imperative inside and outside of health care and does not herself experience defining ‘obesity’ as a medical disease as stigmatizing, rather the contrary. YSD coded the data and conducted the analyses together with SHS and VW. Both are trained as clinical psychologists, but with no experience in the treatment of ‘obesity’, nor experience of living with a larger body themselves. VW has special competence in qualitative methods and narrative interviews, while SHS has extensive experience with qualitative research exploring the client perspective of illness and treatment, with her main clinical training in treatment of complex trauma.

In this paper, we have chosen to mainly use the words “larger bodies,” “higher weight,” or “weight gain” when referring to the phenomenon explored in this study. This is due to many people living with larger bodies finding the English word ‘obesity’ stigmatizing and pathologizing. As we are also discussing the term and diagnosis of ‘obesity’ and how it has evolved and been constructed in the field of medicine, we are using the word ‘obesity’ in single quotation marks in these parts of the paper to show that we are referring to the medical term/condition and not to people. In this study, participants seeking treatment for ‘obesity’ were interviewed and the recent participation in lifestyle treatment for ‘obesity’ was part of the study context and framing. The participants were frequently using the term ‘obesity’ themselves in the interviews. One participant also specifically narrated how he found it stigmatizing that health care professionals seemed to be reluctant to use the word ‘obesity’, while for him this was not a stigmatizing word, highlighting the complexity in choosing appropriate and non-stigmatizing language. It is our experience that our native language term for overweight might be experienced as less stigmatizing than the English words, and not as tied to the medical field, while the native language terms equivalent to “larger bodies” and “fat bodies” might have been experienced as more invasive and stigmatizing in this interview context. The word “overweight” was therefore chosen for some of the interview questions following discussions in our team. In the quotations supporting the results, we are using the terms as expressed by the participants. We use people-first language, “people with larger bodies,” to express that body size is only one of many aspects influencing and constituting identity.

### Data Analysis

Reflexive thematic analysis was used as a framework for analyzing the interview transcripts (Braun & Clarke, 2006, 2019). Upon initial reading of the data material, we decided on an analytical focus exploring the participants’ experience and understanding of their weight development and factors related to weight changes. The analysis was based on Braun and Clarke’s six-phase model and then extended as our initial analysis revealed a need to explore trajectories of experienced weight development. This resulted in an analytical process consisting of seven phases, as detailed below. Phases 1–4 corresponded to phases 1–5 in Braun and Clarke’s model (Braun & Clarke, 2006, 2019). In phase 5, we drew on life story (McAdams, 2008) and narrative perspectives (Riessman, 2008) to formulate dimensions in the data material differentiating trajectories of experienced weight development, and in phase 6 we described and categorized different trajectories. The last phase corresponds to the sixth phase in Braun and Clarke’s (Braun & Clarke, 2006, 2019) framework.

In phase 1, all three authors read the transcriptions of the interviews thoroughly and made notes about ideas for topics, interesting findings, and thoughts. The group met and discussed our notes. One of the aspects of the material that we found interesting in this phase was that several participants did not appear to have any clear-cut explanatory models for their weight gain. They seemed to use the interview to test different hypotheses and ended up rejecting many of these self-proposed models as not relevant to themselves. We decided that when coding the data, we would need to pay special attention to what explanations of weight gain were actually rejected even though they were thematized. We also noticed that most participants highlighted adverse childhood life events when asked about which factors in their history were related to weight gain; however, few explicitly communicated how they experienced the phenomena to be related.

In phase 2, YSD coded the interviews using NVivo 12. The pieces of text interpreted to inform the research question were given code names, and 229 codes were created. In phase 3, following this initial coding of the data material, all authors met to review the codes and search for themes. We used an inductive approach in this part of the data analysis, trying to organize the codes created from the data material into broader more overarching categories based on relevance to the topic researched rather than from a pre-defined theoretical model (Braun & Clarke, 2006). YSD organized the codes into 10 themes with sub-themes based on these discussions.

In phase 4, all authors met again to review and continue to refine the themes. Seven themes and 24 sub-themes were created and named. We re-organized some of the themes identified in phase 3 (see Table 1) and found that the themes created covered the most central content of the meaning-making process of the participants in explaining their weight gain and what maintains it. However, we thought that some important aspects of the participants’ experiences were not covered. This included how they could be placed on the continuum of understanding versus not understanding their weight development and to what degree their weight story revealed that they experienced agency versus no agency concerning weight development. It also became apparent to us that the experienced mechanisms for weight gain that the participants narrated manifested themselves as quite different throughout the life span of the individuals. In phase 5, we therefore decided to read the material again and seek to identify different trajectories of weight development stories based on these two dimensions.

**Table 1.** Thematic Framework: An Overview.

Main Theme	Sub-Theme
1. Always been big	a) Genetic vulnerability
	b) Steady weight gain
2. Poor support for healthy habits in childhood	a) Food as reward or comfort
	b) Poor parental boundary setting
	c) Difficult relationship to food
3. Adverse and traumatic life events in childhood and adolescence	a) Neglect and parental alcohol abuse
	b) Bullying
	c) Too much responsibility and limited emotional support in childhood
	d) Moving a lot in childhood
	e) Parental divorce
	f) Rape
4. Using food for emotion regulation	a) Eats when feeling lonely
	b) Eats when feeling little self-worth
	c) Eats to relax and relieve emotional pain
5. Difficulties with self-care and identity	a) Weight justifies social avoidance
	b) Always prioritizes others before self
	c) Self-criticism
6. Stressful life—no time for eating healthy and exercising	a) Healthy eating needs constant focus
	b) Perfectionism and thought bugs
	c) Work and/or family takes all time and energy
	d) Unsupportive and unsafe relations
7. Unhelpful eating behaviors	a) Portion sizes too large
	b) Unhealthy food choices
	c) High food responsiveness

In phase 6, the research team met again and the 10 weight development stories were organized into four trajectories: (1) Snowballing weight gain—when it starts rolling, I am unable to stop it (understanding/lack of agency), (2) I see the path that leads here—and I may choose another path (understanding and agency), (3) Why me? Grasping at straws (lack of both understanding and agency), and (4) What happened? Making the best of it (lack of understanding but having agency).

Finally, in phase 7, YSD wrote the presentation of the findings and the narratives of the different weight trajectories. SHS and VW reviewed and revised the presentation of findings.

### Ethical Considerations

The project was approved by the Regional Committee for Medical and Research Ethics, Western Norway, and SIKT data protection services. Participation was voluntary, and all participants received both oral and written information about the study and signed an informed consent statement. Participants were informed that they could withdraw their consent at any time. Because the topics of the interviews could be potentially vulnerable, a debrief session was scheduled straight after the interview. Specific risk plans were made if suicidality became a topic. The project was evaluated as posing little risk for the participants, while the knowledge gained could be potentially important for improvement of health care for people with larger bodies.

## Findings

Many of the participants found the health consequences of living with a larger body to be an impediment to living a desired life, for example, not being able to do their work and finding it hard to hike or to play actively with their kids, and described this as a motivation to seek lifestyle treatment. Few reported having experienced direct weight stigma, but most found weight stigma in society to be pervasive. Several participants described a complicated relationship with their bodies and internalized stigma and shame as a hindrance in their everyday life. This could take the form of not going places where light clothing is demanded, avoiding physical intimacy, and putting marriage or other important life events on hold until becoming normal weight.

Through our analysis, we conceptualized the participants’ stories to be structured along two dimensions: degree of understanding and degree of experienced agency regarding weight changes. While some participants had a narrative where they conveyed some clear hypotheses and thoughts about why and how their weight developed during their life history (*N* = 5), others tested several hypotheses searching for an understanding without really arriving at a narrative that matched their lived experiences (*N* = 5). The other dimension, agency versus limited agency included, respectively, four and six of the narratives and describes degree of experienced control concerning weight development.

All trajectories included topics about childhood family values on food and weight, parenting practices, and eating and activity habits. Establishing their own lifestyle habits when moving away from home was thematized in all interviews. Four of the participants particularly highlighted how they started to gain weight rapidly when they moved away from home. Weight gain in this phase of life was presented as either a consequence of a new won feeling of freedom from strict diet rules or a lack of awareness and knowledge about food and cooking.

When asked about life events experienced as related to gaining weight, 8 out of 10 participants told stories about adverse childhood or adolescent experiences such as bullying, death of a parent, neglect, violence, rape, frequent moves, family members having eating disorders, and parental alcohol abuse. Food as emotion regulation was discussed in all trajectories from the understanding end of the continuum. In contrast, the participants in search of understanding highlighted genetic and somatic causes of weight gain, like cessation of smoking or low metabolism. They also described themselves as not paying enough attention to eating and weight or not having the time and energy to be physically active and prepare healthy food.

When analyzing the narratives of the participants, we found that the factors considered pertinent for weight gain in a particular person could change in different stages of their life. As a result, participants could give different meanings to their weight gain depending on which period of life they narrated. For instance, one respondent explained that gaining weight in school age was related to parental eating practices, while gaining weight in young adulthood was caused by experiencing a sexual assault. The latter weight gain was described as an attempt to protect herself from potential future assaults and constitutes a very different understanding of the mechanism for weight gain within the life story of the same individual.

In the following, the story of the four different identified trajectories is narrated: (1) Understanding but limited agency, (2) Understanding and agency, (3) Limited understanding and limited agency, and (4) Limited understanding but experiencing agency.

### Trajectory 1: Snowballing Weight Gain—When It Starts Rolling, I Am Unable to Stop It (Understanding/Lack of Agency), *N* = 3

In this trajectory, the participants provided a clear understanding of how their present higher body weight is related to restricted access to food in childhood and using food for regulating emotions, especially fear, due to childhood neglect and traumatic experiences. This pattern of emotion regulation was described as very difficult to control and change because of the way it was integrated into personality functioning, traumatic memories, and bodily sensations from an early age.

The experience of restricted and variable access to food in childhood was described in slightly different ways. Two of the participants told stories about how they would never know when food was available at home or whether there would be enough for everybody. The variability was due to poor economy, parental mental health problems, and alcohol abuse.

The food was on the table … and if you were slow you got half a potato … if you were fast you might sometimes feel somewhat full … but if you talked you might get so hungry for so long that the next time the food was on the table you made sure to eat as fast as hell … from I was a preschooler I remember this fight for food … it is still following me. I feel afraid of not getting enough … (Participant 3)

In the third narrative in this trajectory, the food restriction was due to parents being preoccupied with dieting. The experienced food restriction in all three cases was understood as causing difficulties in regulating food intake later in life. In the first two cases, the mechanism was described as learning that you must eat when food is available to ensure that you will get enough, while for the third case the forced dieting in childhood was explained as causing a psychological opposition against eating healthy all the time.

In this trajectory, traumatic experiences in childhood and adolescence were described as negatively influencing the feeling of safety, self-worth, and the experience of the body. As a result, the participants described how food became a means of emotion regulation especially when experiencing fear.

The food regulates the bodily tensions … I am afraid … I am still afraid … but I do not know what I fear. It’s all there in my body … it is not possible to get rid of when it’s that old. (Participant 10)

Several participants described their relationship with food as regulation of somewhat sub-conscious reactions to strong feelings of fear and abandoned loneliness. They explained that the internalized and integrated nature of reacting makes it especially difficult to change.

It was comfort and it was also the need to get so full that you got that pain, nausea and numbness … so that you don’t have to feel anything … () It is internalized … I am sad and now I need (food as) comfort. (Participant 9)

For one of the participants, weight gain and a larger body provided a sense of safety and protection after experiencing a sexual assault.

After several years I told my mum that I had been raped … () and she told me that you just have to leave this behind you and move on with your life. But what happened was … I would not admit to myself how I was feeling … and I started to gain weight rapidly … I got depressed … and no one knew. Instead of studying I went home, sat in the dark continually eating … I looked in the mirror and told myself: No one will rape a fat girl. Now I am safe. (Participant 9)

Participants that experienced neglect and violence in childhood described relationships as vulnerable as it was hard to feel safe and valued. Likewise, self-care and confidence were experienced as difficult at times.

I’m in a safe relationship now … and it is really hard to handle. I have never experienced a nice relationship before, and I cannot let the guard down … I expect him to leave me, right? … and then I would like to leave first..(…). He says I am good at treating myself badly, but I am really only repeating what everyone else has done to me over the years..(…). I still often feel lonely and then I eat for comfort…(..). Food is a friend and somewhat the same thing as being social. It provides comfort and if you fail it serves as a punishment. If you succeed it’s a reward. For me food is equivalent to feelings. (Participant 10)

Concretism in describing how the larger body directly communicates emotions, self-worth, or boundaries was present in all the life stories constituting this trajectory. As presented above, the larger body could be experienced as protection against unwanted sexual attention. In contrast, another participant described how his body was experienced as a visual sign of worthlessness and failure that everyone could observe, leaving a sense of intense vulnerability, shame, and feeling unprotected.

If I see someone I know, I turn around … I cannot face the shame of having failed in that way (living with a larger body)..() my body size is the only thing I have been able to measure myself with … I haven’t had anyone that sees me for who I am or that would listen to my experiences. (Participant 3)

One of the participants described how she now feels that her life and self-confidence have improved, but still the emotional eating pattern often feels automatic and hard to break. When having a bad day or feeling stressed, eating for comfort is still easily triggered even though life in general is satisfying.

In this first trajectory, adverse childhood and adolescent experiences were understood as causing an internalized pattern of eating for emotion regulation. Early integration of this pattern and the way it is interwoven with the feeling of self-worth, identity, and relationships makes it hard to change. The body is experienced as communicating directly to others about emotions and personal value, for some a means of protection, while for others a sense of intense vulnerability. Restricted access to food in childhood was seen as causing overeating later in life, either as a protest or an internalized fear of not getting enough food.

### Trajectory 2: I See the Path That Leads Here—and I May Choose Another Path (Understanding and Agency), *N* = 2

In this trajectory, the participants explained weight gain as arising from unhelpful eating habits established in childhood, while being quite physically active. When physical activity decreased in adolescence, weight gain followed. Social difficulties with peers were understood as causing patterns of emotional eating in adolescence. Both participants described that they took on a lot of responsibility with varying support early in life, and in adulthood they have been high performers in work life not prioritizing self-care. However, they now describe themselves as mainly satisfied with their lives, having resolved or come to terms with some earlier emotional challenges and in a phase with more energy and time for self-care.

In this trajectory, food habits in the childhood families were described in terms of food rewards, and availability of food, including flavorful, energy-dense food, without much parental boundary setting. Parents also used food to regulate their relationship with their children, for example, when comforting their children or when feeling guilty for not being a good enough parent. The participants highlighted that as children they did not have the maturity to regulate intake of energy-dense food themselves. This was experienced as causing unhealthy habits concerning sugar and fat intake, portion sizes, regularity of meals, and eating in the absence of hunger.

We got all the food that we asked for … a good dinner every day … and we got candy, cakes and soda, … and crisps … everything that you don’t need … it’s ok once a week, but this was Friday, Saturday, Sunday …. and then Wednesday … I could not stop it myself … I was just a child … I did not manage to turn it down and say that this is not ok..() … My father had a bad conscience and I got sweets, and he could keep on drinking … () the food was a replacement for not being emotionally available and the instability he caused. (Participant 7)

Both participants described themselves as being very physically active during childhood and adolescence, preventing them from rapidly gaining weight.

I did not have a healthy diet, but because I trained 6 times a week with high intensity, I did not gain weight..()..and then when I stopped training I started to gain weight rapidly … going from 5-10 kg overweight to 20 kg. (Participant 2)

Both participants moved several times during their childhood, and even though they experienced some good friendships, they described the effects of moving as a doubled-edged sword. On one hand, the burden of frequently starting on bare ground socially and not having long-lasting friendships was described as hard, but respondents also said that it made them stronger.

It was difficult starting at a new school every second year … you have to change friends, teachers, activities and all of your life … it was hard on me. (Participant 7)

I have moved a lot and I don’t have that much contact with people afterwards … and my relations are mostly tied to areas of performing different tasks together rather than just being friends. (Participant 2)

One of the participants also experienced several adverse life events in childhood including parental divorce, death of a parent, and weight-related bullying. Both described how emotional eating patterns evolved and how they understood this as a means to regulate themselves when facing interpersonal challenges, loneliness, and often putting others’ needs before their own.

I felt like I was not important to anyone … the overweight kept following me because I have always eaten for comfort … to compensate for this or that, moving or new, new, new … It was hard. (Participant 7)

I have realized that I have used my overweight as an excuse or protection from doing things that are a little bit scary … for instance socially … I will do it when I reach this or that weight … but what if I never reach any of those weights?” (Participant 2)

In this trajectory, growing into adult life was associated with performing at high standards in work life, together with putting a lot of value and effort into caring and being helpful to others. Combined, these two aspects were described as leaving little time and energy for self-care and establishing healthy eating and activity habits. Using food as relief in stressful times also continued as an integrated part of everyday life.

I feel that I am there for everyone and everything … I do and give so much to others … and sometimes I feel. Why am I doing this? … I don’t get anything in return. (Participant 7)

I don’t let go of control in any aspect of my life, except from when I am eating. Then I am losing control all the time. (Participant 2)

Regarding the agency dimension in this trajectory, both women described themselves as somewhat having come to terms with their life experiences, working through emotional pain and now feeling satisfied with most aspects of their lives, as well as experiencing a general sense of self-worth. Their psychological processing of earlier life challenges and supportive network at present, together with self-confidence in other areas of life, were seen as important for experiencing agency in changing habits. They expressed now being ready and able to prioritize self-care both in the form of establishing new eating and activity habits and stabilizing their weight, together with finding new strategies for emotion regulation.

I need to give myself more credit and space … everything does not need to be so serious or perfect … I am a quite normal person. (Participant 2)

And I would like to say to myself and all people that are overweight … you need to prioritize yourself … that does not mean that you are selfish … you need to set aside time to care for yourself … buying and preparing the healthy food you need not to gain weight and to stay healthy..() and my daughter supports me and tells me that I want you around for as long as possible. (Participant 7)

In this trajectory, both participants described the use of food rewards, high availability of energy-dense food, and lack of parental help to regulate food intake in childhood. This was experienced as resulting in unhealthy eating habits causing later weight gain. However, the participants convey a general experience of being loved by their parents, despite having to take on a lot of responsibility for themselves from an early age. High performance at school, at work, and in relationships in young adulthood left little time for self-care. When faced with the need for changes in eating and activity habits for health purposes, they now feel able to prioritize this and conduct the changes.

### Trajectory 3: Why Me? Grasping at Straws (Lack of Both Understanding and Agency), *N* = 3

In this trajectory, the participants talked about how they struggled to understand why they have a higher weight. Weight gain was described as somewhat of a mystery, a surprise, and with a quality of being unfair. While preoccupied with not understanding what factors were related to their weight gain, doing something about it was also experienced as difficult. As one of the participants put it, “No matter what I try … It does not work *…*” (Participant 1). In the interviews, a search for possible explanations for weight gain was evident, but many of the formulated hypotheses did not fit with their lived experiences.

The data from the interviews in this trajectory included many statements about not understanding why they gained weight in comparison to others. The participants said that they have always eaten the same as their family or friends, and they describe their eating habits as normal and healthy. Therefore, it is perceived as a mystery why they gained weight while most of their families and friends did not. They explained that they had always been big or steadily gaining weight. These kinds of statements were not present in the other trajectories.

I have always been big … it is not like I suddenly gained weight … As long as I can remember I have been told that I am too big or too fat. (Participant 8)

I have never been an overeater … so why me? … I really don’t understand..()..I never felt that much hunger, and I have always eaten normally … same portions as everyone else … (Participant 1)

In this trajectory, childhood families were described as preoccupied with eating healthily and not gaining weight. In two of the narratives, the mothers had eating disorders and their relationship to body and food was described as influencing their children in different ways. One of the participants developed a bulimic eating disorder during adolescence herself.

My mother was very preoccupied about being thinner … always on a diet … no matter if she was thin … she was always fat in her own head..() ..she was scared and had a really poor self-esteem … and that is what I have grown up with … A mother that did not want me to become like her. (Participant 6)

The trajectory further is characterized by how weight gain was described as developing gradually and somewhat unnoticed.

I did not really notice … but one day while washing the bathroom I just stepped on the weight … and I thought: hot damn … this is way beyond big! … I had not suspected it or thought about it … but afterwards when I looked at older photos it was very obvious. (Participant 8)

I have not really been thinking about it … or cared … it was just how it was … until now when it is a barrier for me at work. (Participant 1)

The hypotheses proposed to explain weight gain in this trajectory consisted to a large degree of genetic and somatic factors. Emotional eating was mentioned but not found to resonate with the lived experiences of the participants.

We have many relatives in our family that are big … it is not just what they eat … it seems to be a genetic family vulnerability. (Participant 6)

I have not been eating often enough … and I have been told that maybe I have been eating too little for too long so that my metabolism was not working as it should be. (Participant 8)

Further, having little time and energy for being physically active and eating healthily was discussed as a barrier to preventing weight gain in adulthood. Maintaining healthy habits was experienced as needing a constant focus and being stricter than others with what you eat.

At that time when I gained weight most rapidly … it was a period when I worked a crazy schedule … 12 to 18 hours every day … my head was totally occupied with work..() I would like to say that it did not influence my eating … I did not think about it at the time … but I think that it maybe did. (Participant 8)

The experience of searching for answers, not fully understanding, and the fact that the weight gain may have a genetic explanation hampers the feeling of agency in this trajectory.

I really don’t know … my mother always made healthy food, I ate what everyone else had … they dragged me out for hiking and activities … but nothing has worked … so now I have really just given up. (Participant 1)

The narrative of the person describing an eating disorder diverged from the others in the descriptions of how food restriction in adolescence and early adult life led to overeating and several cycles of large weight losses on powder diets, regaining weight when starting to eat normal food again. This left the participant feeling out of control when eating normal food, and it was experienced as a mystery why she gained weight while others eating the same meals did not.

In general I eat healthy … but when I try to lose weight I use powder diets that works for me and I may lose 20 kg … and then when starting to eat normal food again … I get on the weight and I see that it goes up … and I think … I have eaten normally today … maybe too big dinner? (Participant 6)

In this trajectory, the participants described a slow and steady weight gain that started at an early age. They struggled to understand why they, in comparison to others, developed a larger body size and conceived their own eating and activity patterns as being normal. The most prominent societal explanations for higher weight like unhealthy eating, not getting enough exercise, or emotional eating were not found to fit with their lived experiences. Participants suggested genetic and physiological factors may be more plausible explanations for their weight gain, leaving them with an experience of limited agency in influencing their weight.

### Trajectory 4: What Happened? Making the Best of It (Lack of Understanding but Having Agency), *N* = 2

In this trajectory, the participants were not very preoccupied with understanding why and how they developed higher weight. As in trajectory 3, genetics and somatic causes were presented as plausible explanations together with not consciously working on establishing healthy habits. Life, relationships, and self-worth were in general described as satisfactory. Establishing healthy habits and attaining weight reduction were in this trajectory experienced as possible, but in need of focus and hard work. The participants described a sense of being able to make a change if they choose to.

Family eating habits in childhood were not broadly described in these interviews, but availability of food and relaxed parenting practices were mentioned together with eating energy-dense school meals. The general family climate was characterized as caring by both participants.

If I asked for some sort of food I would always get it. There were no limits. (Participant 4)

For both participants, having a larger body was not something they related to before they were adults. They described how they did not have a conscious relationship with food, activity, or weight, because other life areas were more important to them, and they were generally satisfied with their lives and social networks.

I have not really been conscious about being big or heavy before recently when I was hospitalized due to high blood pressure and cardiac problems. (Participant 5)

Genetic predisposition, quitting smoking, not being breastfed, and having restricted physical activity due to physical injuries were suggested as hypotheses for gaining weight. The factors understood as causing weight gain in this trajectory were mainly external.

I had too limited physical activity and it was also a genetic predisposition. There is no doubt about that … (Participant 4)

You hear about that false metabolism … that smoking makes your metabolism increase … then you quit smoking and the metabolism goes down … and you start gaining weight like I did. (Participant 5)

In addition, the participants experienced a clear link between being more physically active, improving eating habits, and weight stabilization. Having enough time and energy was described as a necessity for maintaining healthy habits, whereas having more difficult and busy schedules often lead to weight gain. Weight was, to a limited degree, described as linked to emotion regulation in this trajectory.

When the weight goes down it is because I have had time for exercising … and when it goes up I have not been physically active … The motivation is not there all of the time, but now I observe that the weight goes steadily down … so I know that I am actually doing enough. (Participant 4)

I think it is about focus and attitudes towards food, right? Because when I have developed overweight, something must have happened. You have established some unhealthy patterns, and it feels ok. The sofa is nice … and the difference between 3 potatoes and 6 potatoes, does it really matter? And a little bit more sauce because it is tasty, right? (Participant 5)

The participants in this trajectory in general talked about a satisfying social life, good family relations, and a feeling of being competent. Their feeling of competence also generalized to a sense of agency in behavior change, even though they did not fully understand why they gained weight in the first place. For these participants, making healthy changes for themselves was more important in their narratives than trying to understand why they have a larger body.

## Discussion

Narrating about the understanding of weight gain and body size is complicated due to the intertwinement of body size, identity, and sense of self-efficacy, as well as relating to the multitude of theories about weight gain put forward in society. The dimensions of understanding versus not understanding the development of a higher weight and experienced agency versus limited agency in weight changes represented a common thread appearing throughout the narratives. Further, this study found that adverse childhood experiences and food as emotion regulation were the most frequently narrated understandings of weight gain, in addition to lack of support in forming healthy habits when growing up. Stress in adulthood, demanding life situations, and interpersonal challenges were also perceived as influencing eating and physical activity and consequently resulting in weight gain.

We would like to discuss the following questions: Why is it so difficult to narrate about causality in weight gain? How is the balance between individual responsibility and ‘obesity’ as a disease narrated and what consequences do these conceptualizations have for agency and experienced stigma? What role do experienced adversities at a young age play in the narratives about development and maintenance of a higher weight?

### Difficulties of Narrating Weight Gain and Body Size

The participants in our study had recently been seeking weight loss treatment, indicating that they (and/or others) experienced their weight as a problem in need of intervention. Likewise, the interview guide, even though it included quite open questions, also invited discussion of experienced challenges with having a higher weight, framing it as a problem. Thus, the context demanded the participants to provide an explanation for and a narrative about a “problem” that they have been seeking help for together with a person displaying the same “problem” (the interviewer), constituting quite a demanding task. In addition, the “problem” of having a higher weight is a part of the body and could be intertwined with identity and sense of self. A core symptom in eating disorders is the equating of body, weight, and self-worth, giving body size a disproportionately large influence on the definition of identity and self-value (Escandón-Nagel et al., 2024; Fairburn, 2008). People exposed to traumatic experiences are more likely to develop this embodiment of self-valuation (Rodgers et al., 2019). This pattern of embodiment of self-value was also evident in some of the narratives in this study. Being in this position could make it harder to narrate their story about weight gain because it is very closely linked to vulnerability and self-valuation. The experience is not only that having a larger body is a problem, but you are the problem. The “problematic body” becomes both an object and a subject in the narrative (Mol & Law, 2004).

As the participants were very forthcoming in sharing their experiences, they might also have wanted to show that they were knowledgeable about weight and healthy habits, resulting in them exploring many different stories about causes of weight gain rather than arriving directly at their own experiences and opinions. Thus, parts of their narratives had the quality of hypothesis testing rather than experiencing. Some of the participants displayed distress when not being able to reach any understanding of their weight gain that they could resonate with during the interview. This experienced lack of understanding was described both as a frustration because it hampered their sense of agency and because they felt that they were not helping us to gain knowledge in the research project. For the majority of our participants, however, narrating about the function of food or body size throughout their lives appeared to be much more meaningful and important than reflecting on the causality of their weight gain. Ekman (2018) described a similar pattern from qualitative interviews with people with larger bodies in Sweden, where the narratives shifted from behavior focus to descriptions of experienced underlying causes and functions of behaviors. These shifts were understood as helping the participants give meaning to their experiences and providing a more self-compassionate stance, as well as a more socially acceptable explanation for weight gain that could promote emotional support.

### Larger Body Size—How Is the Balance Between Individual Responsibility or ‘Obesity’ as a Disease Narrated?

Interestingly, the people expressing a limited understanding of their weight gain in our interviews, to a large degree, arrived at genetic vulnerability as an explanation, in line with most medical guidelines (Busetto et al., 2024; ObesityCanada, 2024). Putting forward this hypothesis about genetic causes, however, appeared as being the “last option” for participants after discussing several other hypotheses of more individual causation. Our interpretation was that reaching this conclusion about causality was experienced as needing justification and somehow represented not being able to take individual responsibility for one’s health. This could be understood as the participants striving to provide a narrative of personal control and agency, in line with expectations of the lifestyle treatment they had taken part in or to fit with their identity of being competent people, even when this story about their weight did not appear to fit their experiences. These findings illustrate how these narratives are constructed in response to the individual responsibility framing of weight gain and at the same time try to protect against the stigma associated with it. Greener et al. (2010) described a similar phenomenon in their interviews about reasons for gaining weight, finding the participants living with a larger body continually shifted their position between personal responsibility and external factors in a negotiation of causality throughout the interviews. Greener describes these shifts in relational terms as participants negotiating in the interview setting whether they are to “blame” for their weight gain or not (Greener et al., 2010). Imhagen et al.’s (2023) study interviewing people enrolled in a lifestyle ‘obesity’ intervention similarly found that, while asking about their experiences of living with larger bodies, the participants found it imperative to explain to the interviewer why they had gained weight. They discuss this phenomenon as a manifestation of weight stigma in society and health care, prompting participants’ need to justify having a larger body for the interviewer (Imhagen et al., 2023).

Narrating causes of weight gain and body size taps into the question of what degree of personal control of weight is possible (or not). This topic of experienced agency versus limited agency was also evident throughout the participants’ stories in the form of narrative negotiation. Our findings show that participants placed themselves differently on the continuum of agency. Experience of agency was related to a general sense of competence and self-worth, support, and having processed or come to terms with negative life events and emotional pain. As a result of this, the function of food in their lives changed. Agency was mainly formulated in connection to establishing healthy habits and self-care in the participants’ narratives, but also related to hopes for long-term weight changes. While establishing healthier habits and strategies for self-care could be considered viable outcomes of behavioral lifestyle treatments and are associated with improved quality of life (Marcos-Delgado et al., 2021; van Dammen et al., 2018), research finds it highly questionable whether long-term maintenance of weight loss is a realistic outcome (Nordmo et al., 2020). Linking agency to weight changes rather than to behaviors or quality of life could therefore be considered counterproductive in ‘obesity’ care, setting many people living with a larger body up for a feeling of failure and thereby contributing to stigma. The lack of agency in some of our participants’ narratives was described as related to repeated failures of weight loss attempts, leading to self-judgment and shame, in line with the criticism of lifestyle interventions raised by critical fat scholars (Ekman, 2018; Ferreira & Webber, 2022). Interestingly, the authors could not find literature discussing this type of lack of agency and self-blame in lifestyle interventions for other types of chronic diseases. Several of the aspects discussed might contribute to this difference, for instance, health care professionals communicating that body size could be entirely reversed by willful effort, weight reduction as the main goal of treatment, the intertwinement of body size and identity, the visibility of the larger body, and the stigma against larger bodies in society. Our study also found that few, if any, of the participants interviewed perceived ‘obesity’ to be a chronic relapsing brain-based disease of appetite regulation in line with definitions in medical guidelines and stakeholder organizations (Busetto et al., 2024; ObesityCanada, 2024), indicating that knowledge of what mechanisms constitute ‘obesity’ as a chronic disease is not widely communicated, accepted, or informing health care. Further, that weight stigma and the individual responsibility framing of body size still represent the main societal narratives about larger bodies shaping the experiences and narratives of our treatment-seeking participants.

### Childhood Adversities and Weight Gain

Our findings that adverse childhood experiences, neglect, and eating for emotion regulation were the most prominent explanations for weight gain are in line with the meta-synthesis from Farrell et al. (2021). Quantitative data also support that childhood trauma has a major role in the development of higher weight both in childhood and later in life (Hemmingsson et al., 2014; Schroeder et al., 2021). Some of our participants talked about how their sense of agency in eating and avoiding weight gain was hampered by the strength of emotional trauma reactions, despite understanding the relationship between traumatic experiences and weight gain. They were describing the important function of eating in avoiding or regulating emotions in their daily life.

Severe childhood traumatic experiences were also linked to experiencing the large body as a visual sign of not being worthy of love or being a failure, leaving the people feeling extremely vulnerable and exposed. This embodiment of self-value is also theorized as the core symptom of eating disorders (Fairburn, 2008; Hrabosky et al., 2007) and might be part of the pathways from higher weight to developing an eating disorder. Interestingly, most lifestyle treatments are based on the principles of behavioral therapy and do not directly address trauma reactions, internalization of weight stigma, or emotion regulation. Thus, there seems to be a mismatch between many of our participants’ experience of their own weight development, what maintains weight gain, their needs, and the rationale of the treatment offered.

## Implications for Practice

Unrealistic expectations about long-term weight loss following behavioral treatment might make people participating in these interventions blame themselves for not being in control or working hard enough, contributing to stigma. As the narratives about causes for weight gain and the realistic outcomes of different interventions to a large degree are created together with health care professionals when seeking treatment (Luig et al., 2020), health care providers need to empower the people with larger bodies seeking health care in creating narratives that are free of blame and hold realistic expectations of change. There might also be a need for discriminating between what causes ‘obesity’ and what could be targeted in ‘obesity’ care for providing the best possible life despite biological vulnerabilities and experienced adversities. As narratives centered largely around individual responsibility for weight gain, either as failure to adhere to healthy habits or not being able to because of emotional pain, health care professionals might need to spend more time explaining the biological basis and physiological mechanisms for ‘obesity’ in a way that makes it integrated for the people seeking ‘obesity’ treatment (Grannell, le Roux, et al., 2021). A focus on altering the environment and the available support of individuals could also be a helpful approach in health care, reducing stigma.

Assessing and addressing traumatic life experiences and food as emotion regulation in health care for people with higher weights stands out as particularly important. The participants in our study who understood their weight gain in light of trauma and emotion regulation consisted of a subgroup that had received previous help for their trauma experiences and a group with ongoing trauma reactions and internalizing symptoms. While the subgroup that had somehow processed their trauma experienced agency in creating a good life for themselves, the latter experienced lack of control over behaviors and emotions. This might indicate a need for more trauma-oriented treatment, without focusing on lifestyle habits until emotional trauma reactions are more stabilized. Viewing ‘obesity’ as a chronic, relapsing disease, the follow-up also needs to be long-term and holistic. This would include a broader definition of health than just weight status and related medical complications and integrate mental health and quality of life.

While this study took place before the new generation of ‘obesity’ drugs were widely available, stories from patients treated with these drugs might shed light on how the physiological changes produced by the medications are experienced to influence hunger and appetite, eating habits, sense of control, and agency, thus creating new narratives about the causality of weight gain. Treatment with medications, together with behavioral interventions, could match the biological understanding of weight gain. However, while ‘obesity’ drugs reduce appetite for most people and lead to more control of eating and weight loss, the medications are not likely to reduce the emotional pain resulting from trauma or internalized stigma, even when emotional eating is reduced.

## Methodological Reflections

The interview setting is a relational experience between a participant and a researcher (Warin & Gunson, 2013). Narratives are co-created and formed by the meeting of the two individuals with their physical manifestations, engagement with different topics, and relational capacities. The interviewer in this study presented herself as independent from the treatment setting at the start of the interview and as having experiences from developing and living with “overweight” herself. This might have created a setting where the difference in weight between the participant and researcher was not “an elephant in the room” or a silenced power structure (Warin & Gunson, 2013). However, even though both the interviewer and participant presented with a larger body, there could still be differences in weight influencing the narrating process in different ways, for example, in creating a safe setting with less inherent power structures or adapting the narrative to avoid hurting the interviewer. When conducting and listening to interviews, our impression is that using the word “overweight” was not getting in the way of respectful communication.

Societal explanations for weight gain were scarce in our material, probably because the interview guide was directed toward the individual’s life stories. Recent participation in lifestyle treatment might also have made individual explanations at the top of mind. Still, in contrast to some earlier qualitative studies (Grannell, le Roux, et al., 2021; Greener et al., 2010), few of our participants narrated their larger body as mainly a result of their own shortcomings. Some described that not feeling able to control weight, food intake, or emotions often made them self-critical and shameful, but most of the narratives focused on experiences with parents and upbringing, lack of safety in their close surroundings, negative life events, stress, interpersonal challenges, and genetics. The Life Story Interview format might have facilitated these long-term, interpersonal perspectives on weight development, rather than a here-and-now focus that might have fostered a more individualized perspective. Several of the participants said that they had never talked through their weight history in a life perspective before and that this was a helpful exercise, having a shame-reducing function.

The life story format of the interviews made it possible to analyze explanations for weight gain in different phases of life. Some stories conceptualized quite different mechanisms for weight gain in childhood versus later in life. One story told about parenting practices as causing weight gain in childhood, opposition to parents as causing weight gain in adolescence, while weight gain in adulthood was understood as a protection against sexual assaults following a sexual trauma. These stories highlight both that factors perceived as related to development and maintenance of a higher weight could differ throughout life, and that body weight and eating might have several different functions and attributed meanings in one individual.

The interviewer being a psychologist might have primed the informants to focus more on psychological and individual topics in the interview. In addition, the interviewer was probably more prone to explore psychological topics and being comfortable with emotions being activated in the room when exploring vulnerable topics. The team conducting the analyses also consisted of psychologists, potentially influencing our interpretation of findings in a more psychologically minded direction.

Interviewing participants who all participated in lifestyle treatment would also influence the results. For instance, participants referred to hypotheses about ‘obesity’ development which they had recently been presented at the lifestyle treatment at the hospital. Still, many people with larger bodies would have experiences of contact with health care services, and the study provides findings that are probably of broader relevance. The project, the interview site, and the interviewer were, however, not related to their treatment and therefore would not threaten their alliance with the treatment provider or have any consequences for their treatment. However, the first author having worked as a treatment provider in the eating disorder field for several years would also have influenced the analysis of the narratives due to an interest in how to best meet and support people with larger bodies seeking treatment, but at the same time framing ‘obesity’ as a potential problem in need of support.

The participants were not asked about gender identity or sexual orientation. This could represent a weakness of the study, as these phenomena might be relevant when narrating about body and weight. However, no one mentioned those topics while asked to narrate freely about their weight development in a life-course perspective. All participants interviewed were from the majority ethnic group in our country, limiting the creation of knowledge about the experiences of minority ethnic groups.

## Conclusion and Implications

People living with larger bodies find it hard to narrate the causes of their weight development, and few understand ‘obesity’ as being a chronic brain-based disease of appetite regulation, as described in prominent medical guidelines. The sense of agency in creating a good life and healthy habits was to a large degree related to psychological and interpersonal factors in the narratives. Unprocessed and internalized childhood trauma was experienced as interfering with sense of agency and leading to embodiment of self-value. The sense of agency was also impacted by repeated failure of weight loss attempts, indicating the importance of health care providers to convey realistic and holistic long-term goals in health care for people with larger bodies, as well as using a trauma-informed approach.

## Appendix A

### Interview Guide

#### Introduction

In this interview, I am interested in learning about your life story, and what factors you think have influenced your weight development throughout life. The interview will, of course, not be able to cover all important aspects of your life story and experiences, but we will invite you to focus on some important experiences and topics. There are no right or wrong answers, and we are interested in your personal experiences. I will guide you through the interview and it will last for about three hours.

This is not a clinical assessment or a therapy session. Our goal is to listen to your story and how you give meaning and importance to situations, factors, and experiences from your life. You decide for yourself what information you would like to share or not share, and the information will be treated confidentially and in line with your consent to participate in this research project. The interview will ask questions about some potentially sensitive topics from your life story, but you must feel free to stop the interview or choose not to answer particular questions. Choosing to do so will not have any negative consequences for you.

#### Do You Have Any Questions Before We Start?

##### Development

First, I would like to ask if you could tell me about your weight development and your development of overweight. How did you experience your weight gain and your weight development throughout life? What do you perceive as important factors or experiences that could have contributed to gaining weight? Please feel free to share your thoughts, feelings, or how you have related to your weight development in different phases of your life.

##### Maintenance

Could you tell me about how you relate to your weight now? What thoughts, feelings, and attitudes do you have about yourself and your weight? What do you consider important factors influencing your weight now?

##### Turning Points

Could you identify turning points for your weight development? Is there a specific point in time, something that happened, or something completely different that you consider a turning point? If you already told me about this turning point, would you be willing to describe and explore this in more detail? If you cannot think about any turning points, I would like you to share your thoughts about why you experience it like there have been no turning points.

##### Challenges

If you look back on your life until now, could you describe what you consider to be your biggest challenge? What is or was the problem? How has this challenge developed? How do you relate to/manage or deal with this challenge?

##### Defeats, Disappointments, and Regrets

All people experience defeats, disappointments, and regrets in their lives. Could you tell me about what you consider to be your largest defeat or regret in life? It could encompass any aspect of life. Describe how the situations evolved, how you have tried to deal with it, and how it has affected your life story.

##### Self-Perception

How will you describe your thoughts and feelings about yourself? Feel free to explore in depth. Are any of your thoughts and feelings about yourself influenced by your weight, or how you relate to your weight or weight management experiences?

##### Health and Weight

Try to describe how you experience your life right now. Do you experience any challenges associated with having overweight? Describe to what extent and in what way thoughts about your body, eating, activity, or weight influence your life. Could you talk about your encounters with the health care system (obesity treatment or care)?

##### Message

Is there something you would have liked others to know about overweight/obesity? This could be people that have not experienced living with overweight, health care workers, people who are close to you, politicians, or society in general. Do you have a message that you want to convey?

##### Prospects

What thoughts do you have about your future? (In general and related to weight and health).

##### Overarching Theme

If you should summarize your life story, with all situations, people, challenges, and highlights, could you think of an overarching theme, message, or idea? What is your main topic from your weight story? Could you explain more about the background of your answer?

##### Other Aspects

Are there any important aspects of your life story that I have not asked you about and that you would like to talk about? Or anything else you would like to say?

##### Reflection

Thank you so much for participating in this interview! I have only one last question before we end. I would like you to take a few minutes to reflect on and summarize in short what you consider most important in your weight story (development, maintenance, and management of weight). Do you have any questions or closing remarks?
